# Altered distribution of chronicity-deficient Pseudomonas aeruginosa mutant guides optimization of synthetic chronic wound model

**DOI:** 10.1099/mic.0.001719

**Published:** 2026-07-28

**Authors:** Bethan Roberts, Claire S. Laxton, Daniella L. Spencer, Ida Thaarup, Stephan Heeb, Christopher N. Penfold, Kim R. Hardie

**Affiliations:** 1 Biodiscovery Institute, School of Life Sciences, University of Nottingham, Nottingham, UK; 2 Department of Immunology and Microbiology, Costerton Biofilm Centre, University of Copenhagen, Copenhagen, Denmark

**Keywords:** arginine, biofilms, chronic wound, clinical isolates, virulence

## Abstract

*Pseudomonas aeruginosa* is commonly isolated from chronic wounds where it forms biofilms that are recalcitrant to current treatments, greatly impacting the morbidity and mortality of infected patients. Since *P. aeruginosa* dictates wound severity and treatment outcomes, this bacterium is an important target in the treatment of chronic wound infections. This study aimed to unravel the functional intricacies of a key virulence factor, the arginine-specific aminopeptidase of *P. aeruginosa* (AaaA), which is crucial for the establishment and persistence of chronic infections. While interrogation of genomes revealed that *aaaA* is highly conserved, its activity varied between chronic wound clinical isolates. To explore the phenotypic impact of AaaA in chronic infections, a collagen-based synthetic chronic wound (SCW) model was optimized to enable reproducible assessment of AaaA activity. AaaA activity was increased within the SCW compared to planktonic cultures, further supporting the hypothesis that this virulence factor is integral to chronic infections caused by *P. aeruginosa*. It was also shown that AaaA contributed to biofilm formation as well as providing a survival advantage within the SCW model. Overall, a novel link between AaaA and biofilm formation was verified and served to guide the optimisation of a realistic chronic wound infection model.

## Introduction

Within healthcare, biofilms are key to the establishment of persistent, hard-to-treat infections and the contamination of medical devices and implants. *Pseudomonas aeruginosa* infections are particularly serious in immunocompromised patients, e.g. human immunodeficiency virus and burn patients, where *P. aeruginosa* can cause severe microbial infections leading to a delay in recovery and even death [1]. The bacterium is one of the most frequently isolated pathogens from wound infections such as diabetic foot ulcers, and due to the ability of *P. aeruginosa* to form biofilms, many of these infections progress to chronic states [2]. These chronic infections are becoming increasingly difficult to treat due to the acquisition of new determinants of antimicrobial resistance alongside intrinsic resistances to several antibiotics already possessed by *P. aeruginosa*, meaning clearance of the bacteria via antibiotic therapy is becoming very difficult [3]. Although there is no universally accepted definition for chronicity, a chronic wound is usually identified as a wound that does not progress through the healing stages and is instead held in the inflammatory phase for longer than 2 weeks [4]. With an ageing community, chronic wounds affect 1–2% of the population, with the common types including diabetic ulcers, vascular ulcers and pressure ulcers [5]. Key species isolated from chronic wounds include *P. aeruginosa*, *Staphylococcus aureus*, coagulase-negative staphylococci, *Enterococcus faecalis* and *Proteus* species [2]. Interestingly, using imaging techniques *P. aeruginosa* has also been found in chronic wound samples, which have previously been categorized as culture-negative [6]. This implies that the incidence of *P. aeruginosa* infection within wounds may be higher than previously thought. The prevalence of *P. aeruginosa* within a wound is linked to prognosis, as the presence of the bacterium is associated with more severe and clinically persistent infections and prolonged healing times [7, 8]. Because of this, targeting *P. aeruginosa* within wounds with clinical interventions could improve treatment outcomes.

l-Arginine is an important amino acid in *P. aeruginosa* aerobic and anaerobic metabolism. It allows metabolic flexibility as the scavenged l-arginine can be used as a source of carbon or nitrogen for energy production [9]. As *P. aeruginosa* has evolved several different pathways to produce ATP from l-arginine, the sequestration of arginine from the environment, both aerobically and anaerobically, is very important for both the pathogenicity and overall survival of the bacterial species. Moreover, it has also been demonstrated that l-arginine may have a role in cell signalling and thus regulate bacterial lifestyle, along with modulating the host immune response [10, 11]. Arginine could also provide an advantage to biofilm formation, helping to promote colonization of the bacteria, as well as providing a degree of resistance to the host immune system [12].

AaaA (PA0328), a classical autotransporter of *P. aeruginosa*, has been demonstrated to have arginine-specific aminopeptidase activity. Through a phenotypic comparison between *P. aeruginosa* PAO1L and a defined PAO1L Δ*aaaA* mutant, it was determined that one of the functions of AaaA is to cleave N-terminal arginine from peptides [13]. The PAO1L Δ*aaaA* mutant showed reduced respiration when dipeptides and tripeptides with an N-terminal arginine were the only source of nitrogen and carbon. The effect of AaaA *in vivo* was assessed using mouse wound infection models [13]. These experiments showed no difference in virulence between the parental *P. aeruginosa* strain PAO1L and its derived PAO1L Δ*aaaA* mutant in an acute infection mouse burn wound model. However, in a chronic infection, there was a significant reduction in the survival of the Δ*aaaA* mutant at both 2 and 8 days post-infection [13]. Changes in the immune response were also observed with the Δ*aaaA* mutant, in addition to the variations in bacterial load. Expression of the pro-inflammatory cytokines TNF*α* and IL-1*α* was reduced in the Δ*aaaA* mutant at 2 days post-infection, suggesting that AaaA has a proinflammatory role in acute infection [13]. Expression of inducible nitric oxide synthase (iNOS) and arginase I and II from the host immune system was also lower in PAO1L Δ*aaaA*-infected mice at 2 days post-infection. However, at 8 days post-infection, which is considered a chronic stage of infection in mice, iNOS expression was raised in Δ*aaaA* mutant-infected mice, while arginase I levels fell compared to wild-type PAO1L-infected mice. Interestingly, at 8 days post-infection with the Δ*aaaA* mutant, lower bacterial loads were observed, but increased fibroblast infiltration was seen compared to the wild-type [13]. This implies that, in the chronic stages of infection, AaaA disrupts wound repair and the healing process by potentially disturbing the arginine-regulated immune response [14]. As AaaA is surface displayed, highly conserved within *P. aeruginosa*, and immunogenic, it has potential as a drug target in the treatment of *P. aeruginosa* infections. Because of these characteristics, it was identified as one of the top five potential proteins in a vaccine-target candidate study for *P. aeruginosa*, further demonstrating its therapeutic potential [15].

This study aimed to build upon previous work conducted on AaaA by exploring the function of AaaA in *P. aeruginosa* infection. To do this, a novel chronic wound biofilm model was optimized and utilized, along with the use of clinically isolated strains, to study AaaA in a disease-relevant background. Additional exploratory experiments with a cystic fibrosis (CF) isolate in artificial sputum medium are included to assess whether AaaA-related biofilm phenotypes might also be observed in other chronic infection contexts. Understanding the role and importance of AaaA in *P. aeruginosa* chronic infection could provide a potential therapeutic target and alternative treatment options to existing antibiotics.

## Methods

### Bacterial strains and media

The bacterial strains and plasmids used in this study are detailed in Tables 1 and 2, respectively.

**Table 1.** List of strains used in this study

Organism	Description	Source
*E. coli* S17-1 λ-*pir*	Cloning strain used for conjugations. Genotype: *Thi, pro hsdR-, hsdμ+, recA; RP4 2- Tc::Mu- Kn::Tn7*	[31]
*P. aeruginosa* PAO1L	*P. aeruginosa* PAO1 Lausanne sub-strain	[32]
*P. aeruginosa* PAO1L Δ*aaaA*	PAO1L with an in-frame deletion of *aaaA* (PA0328)	This study
*P. aeruginosa* CW2 B1 (bone)	Isolated from a chronic wound infection via bone biopsy. Intermediate resistance to aztreonam	[33]
*P. aeruginosa* CW3 T1 (tissue)	Isolated from a chronic wound infection via tissue biopsy. Intermediate resistance to aztreonam	
*P. aeruginosa* CW4 T1 (tissue)	Isolated from a chronic wound infection via tissue biopsy. Resistant to ciprofloxacin. Intermediate resistance to aztreonam	
*P. aeruginosa* CW5 B1 (bone)	Isolated from a chronic wound infection via bone biopsy. Resistant to ciprofloxacin. Intermediate resistance to aztreonam	
*P. aeruginosa* CW5 S1 (blood)	Isolated from a chronic wound infection via a blood sample. Resistant to ciprofloxacin and piperacillin-tazobactam. Intermediate resistance to aztreonam	
*P. aeruginosa* CW6 T4 (tissue)	Isolated from a chronic wound infection via tissue biopsy. Resistant to gentamicin, piperacillin-tazobactam, ceftazidime, aztreonam and meropenem. Intermediate resistance to ciprofloxacin	
*P. aeruginosa* CW7 T1 (tissue)	Isolated from a chronic wound infection via tissue biopsy. Intermediate resistance to aztreonam and meropenem	
*P. aeruginosa* CW2 B1 Δ*aaaA*	CW2 B1 with an in-frame deletion of *aaaA* (PA0328)	This study
*P. aeruginosa* CW4 T1 Δ*aaaA*	CW4 T1 with an in-frame deletion of *aaaA* (PA0328)	
*P. aeruginosa* CW5 B1 Δ*aaaA*	CW5 B1 with an in-frame deletion of *aaaA* (PA0328)	
*P. aeruginosa* PAO1L Δ*aaaA* miniCTX::*aaaA*	PAO1L Δ*aaaA* chromosomally inserted complementation mutant. *aaaA* with the native promoter was reinserted at the miniCTX site. Tetracycline resistant (125 µg ml^−1^)	
*P. aeruginosa* CW2 B1 Δ*aaaA* miniCTX::*aaaA*	CW2 B1 Δ*aaaA* chromosomally inserted complementation mutant. *aaaA* sequence from PAO1L with the native promoter was reinserted at the miniCTX site. Tetracycline resistant (125 µg ml^−1^)	
*P. aeruginosa* CW4 T1 Δ*aaaA* miniCTX::*aaaA*	CW4 T1 Δ*aaaA* chromosomally inserted complementation mutant. *aaaA* sequence from PAO1L with the native promoter was reinserted at the miniCTX site. Tetracycline resistant (125 µg ml^−1^)	
*P. aeruginosa* CW5 B1 Δ*aaaA* miniCTX::*aaaA*	CW5 B1 Δ*aaaA* chromosomally inserted complementation mutant. *aaaA* sequence from PAO1L with the native promoter was reinserted at the miniCTX site. Tetracycline resistant (125 µg ml^−1^)	
*P. aeruginosa* LESB65	Liverpool epidemic strain subtype 65	[34]
*P. aeruginosa* LESB65 Δ*aaaA*	LESB65 with an in-frame deletion of *aaaA* (PA0328)	[35]

**Table 2.** List of plasmids used in this study

Name	Description	Source
pEX18-Gm::Δ*aaaA*	pEX18-Gm vector containing 618 bp upstream plus the first and last three codons of the *aaaA* ORF and 617 bp downstream, inserted at the *XbaI*, *BamHI* and *EcoRI* sites via restriction digest. Used to create an in-frame *aaaA* deletion mutant with sucrose selection. Gentamicin resistant (10 µg ml^−1^ in *E. coli*, 20 µg ml^−1^ in *P. aeruginosa*)	This study
miniCTX::*aaaA*	miniCTX-1 [36] with PAO1L *aaaA* ORF plus promoter region (500 bp upstream) inserted via restriction digest at *Not*I and *Eco*RV. Tetracycline resistant (10 µg ml^−1^ in *E. coli*, 125 µg ml^−1^ in *P. aeruginosa*)	[13]

Unless stated otherwise, growth of all bacterial species was in lysogeny broth (LB), with the addition of antibiotics for plasmid maintenance as detailed in Table 2. A list of all media used in this study is detailed in Table 3. Bacteria were routinely grown at 37 °C, 200 r.p.m. in LB for liquid culture or at 37 °C statically for growth on agar plates. Bacterial growth in liquid media was assessed using optical density or absorbance at a wavelength of 600 nm (OD_600nm_).

**Table 3.** List of media used in this study

Media	Use	Preparation
**LB**	Routine Culture	Made according to manufacturer’s instructions (Invitrogen) then autoclaved
***Pseudomonas* Isolation Agar (PIA**)	Isolation of *P. aeruginosa* from a mixed culture	Made according to manufacturer’s instructions (Merck Life Science UK) then autoclaved
**TYS10 agar**	Sucrose selection	Made in house according to [37] then autoclaved
**Minimal media P (MMP**)	l-Arginine-*p*-nitroanilide cleavage assay	Made in house: 10.4 mM Na_2_HPO_4_, 4.8 mM KH_2_PO_4_, 1.7 mM MgSO_4_, 6.6 µM FeSO_4_ dissolved in deionised water and filter sterilized
**Synthetic wound fluid (SWF**)	For use in the SCW model	50% (v/v) FBS (Gibco), 50% (v/v) peptone water (Oxoid)
**Artificia**l **sputum media** (**ASM**)	For use in the Bioflux^™^ model	Also referred to as SCFM and made according to [38]

### Bioinformatic analysis

The sequence of AaaA from PAO1 was used to run a diamond blastp search at https://www.pseudomonas.com/ using stringent (*e*-value cutoff: 10^−12^; default sensitivity, query coverage: 100%, identity cutoff: 95%) parameters on all complete *P. aeruginosa* genomes. The search produced 3,748 hits having between 0 and 27 mismatches. A temporary fasta file was produced with the hits obtained, and unique sequences were extracted using a Perl script, RemoveRep2.pl (Fig. S1, available in the online Supplementary Material). The resulting final fasta file contained 50 unique sequences. Occurrences of each of these sequences in the temporary file were counted (using the batch CLI command grep -wc ‘sequence’ temp.fast >>output.txt). clustal omega was run with the sequences in the final fasta file to produce a cladogram, which was then further manually edited to add the sequence counts.

### l-Arginine-*p*-nitroanilide cleavage (AaaA activity) assay

AaaA activity assay was conducted as stated in Luckett *et al.* [13]. However, 100 µl of the whole-cell suspension was added to a clear 96-well plate along with 100 µl of 1 mM l-arginine-*p*-nitroanilide (Sigma-Aldrich) and placed in a Tecan SPARKs plate reader at 37 °C for 24 h, with absorbance readings at both 410 nm (yellow) and 600 nm (OD_600nm_) every 15 min.

### Gene knockout and complementation cloning

Primers (Table 4) were designed to amplify the regions ~600 bp up and downstream of *aaaA*, including the first and last codons of *aaaA* to create an in-frame, non-functional four-amino-acid-long peptide, and add the restriction sites *Xba*I, *Bam*HI and *Eco*RI (Δ*aaaA*-Up/DnF/R) to each fragment for cloning. PCR was conducted on extracted PAO1L gDNA using Q5 high-fidelity 2X master mix in a total volume of 25 µl, according to the manufacturer’s instructions. Following cleanup using the Monarch PCR cleanup kit New England Biolabs (NEB), the amplicons (Fig. S2a) and *pEX18Gm* empty suicide vector were cut using double restriction enzyme digests in 20 µl reaction volume including >1 µg target DNA, 0.5 µl of each restriction enzyme (all NEB HF, 20,000 U ml^−1^) and (for vector digest) 10 units (1 µl of 10,000 U ml^−1^) calf intestinal alkaline phosphatase to prevent recircularization. The reactions were incubated at 37 °C for 1 h, and then, to improve efficiency, an additional 0.5 µl of each restriction enzyme was added. The reaction was incubated for a further 1 h and then heat-inactivated at 65 °C for 20 min. Following digestion (Fig. S2b), DNA was size separated by agarose gel electrophoresis, visualized on a UV transilluminator, excised using a scalpel and extracted using the Monarch^™^ gel extraction kit, according to the manufacturer’s instructions. DNA quality and quantity were checked using a NanoDrop ND-1000 spectrophotometer (Nanodrop Technologies). Ligation reactions were conducted in a total volume of 20 µl using 1 µl T4 DNA Ligase (NEB, 400,000 U ml^−1^) and 20–100 ng of the insert and vector at a range of ratios (1:2, 1:3, 2:3 of vector/insert). The reactions were incubated in a thermocycler at 16 °C for 16 h and then heat-inactivated at 65 °C for 10 min. The product was dialysed by dispensing the full volume on an MF-Millipore^™^ 0.22 µM MCE Membrane (Merck Life Science UK Ltd.), floating on nuclease-free (NF) water and incubating for 5–15 min. The ligation product was then aspirated and immediately transformed into electrocompetent commercial DH5α *Escherichia coli* by electroporation (NEB). Transformants were plated onto selective blue/white screening plates. Colony PCR of selected white colonies was used to confirm insertion (Fig. S2c) in 20 µl GoTaq Green Hot-Start reactions, with an extended 10-min denaturation step (Promega, UK). Plasmid was extracted from PCR-positive samples using NEB miniprep kits, and the cloning region was sequenced by Sanger sequencing to confirm successful cloning of the pEX18-Gm::Δ*aaaA* suicide plasmid. The plasmid was further transformed into the electrocompetent S17-1 *λ-pir* strain by electroporation. Conjugation was conducted through puddle mating as stated in Hmelo *et al.* [16]. One hundred microlitres of donor recipient mix was spread onto selective *Pseudomonas* Isolation Agar (PIA) plates (BD Difco™) with appropriate antibiotics, and the plates were dried thoroughly and then incubated at 37 °C overnight. For gene knockout, sucrose selection occurred on TYS10 agar, with further selection on PIA plates with the appropriate antibiotic. Whole-genome sequencing (WGS) was conducted by MicrobesNG to confirm successful gene knockout, and genome sequences, both WT and Δ*aaaA*, were uploaded to Bacterial and viral bioinformatics resource center (BV-BRC) (https://www.bv-brc.org/searches/GenomeSearch), genome IDs: CW2 B1 287.5889, CW4 T1 287.5885, CW5 B1 287.5881, CW2 B1 Δ*aaaA* 287.31124, CW4 T1 Δ*aaaA* 287.31125 and CW5 B1 Δ*aaaA* 287.31126.

**Table 4.** List of primers used in this study

Purpose	Name	Sequence	Source
Cloning pEX18-Gm::Δ*aaaA*	Δ*aaaA*-UpF	atattctagaAGGCCATCGAGTACATCAGCCG	This study
	Δ*aaaA*-UpR	atatggatccTTCTGAGCGGGCCAGGGG	
	Δ*aaaA*-DnF	atatggatccGAACACGGCACTTTCCTCTGTGTC	
	Δ*aaaA*-DnR	atatgaattcATCTGAAGAAAGCGAAAGACGGCC	
Reverse transcription quantitative PCR	rpoD-F	GGGCGAAGAAGGAAATGGTC	[39]
	rpoD-R	CAGGTGGCGTAGGTGGAGAA	
	rpoS-F	CTCCCCGGGCAACTCCAAAAG	
	rpoS-R	CGATCATCCGCTTCCGACCAG	
	*aaaA*-F	GTCACCGGCGACAAGATGTA	This study
	*aaaA*-R	CAGGATCTGTTCGCGGTAGG	

### Synthetic chronic wound model

The synthetic chronic wound (SCW) model was made in either 96-well plates or 8-well Ibidi chambers for microscopy. Bacteria for inoculation into the SCW model were grown overnight and then inoculated at 1% v/v into 5 ml fresh LB, which was incubated at 37 °C, 200 r.p.m. until mid-late exponential phase (OD_600nm_ 0.6–0.9). Bacteria were washed in PBS and normalized to an OD_600nm_ of 0.1 in 1 ml synthetic wound fluid (SWF) (Table 3). The SCW was prepared on ice, under sterile conditions. To make a 100 µl volume, which was used in a 96-well plate, 20 µl rat tail collagen (Corning^™^ Collagen 1, high concentration, rat tail from Fisher Scientific) at a final concentration of 2 mg ml^−1^, 10 µl 0.1% acetic acid and 50 µl of the normalized bacteria in SWF were added along with 10 µl of SWF media. Ten microlitres of 0.2 M sodium hydroxide was added to initiate polymerization of the collagen and then aliquoted into the plate, in triplicate. The volumes were adjusted to make a 200 µl model when using an 8-well chamber. The plate was then placed in a humidity chamber, which had been UV (8-watt 254 nm) sterilized for 20 min, and incubated at 37 °C.

### Homogenization of the SCW model and colony-forming units

One hundred microlitres of 500 mg ml^−1^ collagenase was added to each well containing the SCW model, and the plate was incubated at 37 °C for 1 h. Samples were then moved to 1.5-ml Eppendorf tubes, briefly vortexed and incubated again at 37 °C for 30 min. For c.f.u., 20 µl of each sample was serially diluted, and 10 µl samples were plated, in triplicate, on LB plates and incubated at 22 °C overnight until colonies were visible to count. For the AaaA activity assay, the remaining homogenized biofilm was spun and washed in PBS three times, before resuspension in minimal media P (MMP). One hundred microlitres was added to the AaaA activity assay as described above.

### Bioflux biofilm model

Overnight cultures were washed twice, resuspended in artificial sputum media (ASM) and normalized to an OD_600nm_ of 0.05. The ASM contained SYTO-9 and propidium iodide (PI) to final concentrations of 1 µg ml^−1^ and 4 µg ml^−1^, respectively. One hundred microlitres of media was added to the inlet well of a 48-well Bioflux plate (Fluxion Biosciences). The plate lid (sterilized by swabbing with ethanol) was then screwed on using the provided screwdriver. The plate was attached to the Bioflux^™^ 200 pump system (Fluxion Biosciences) by the capillaries for priming. The software Fluxion was used to programme the Bioflux, and the shear was set to 5 dyne cm^−2^ for 5 min or until the inner circle in the outlet was filled with media. The associated temperature control platform was set at 37 °C and remained at this temperature for the duration of the experiment. The plate was then disconnected from the capillaries, the lid was removed and 70 µl of normalized culture was added to the outlet well. The media was added to a total of 1,200 µl in the inlet well, and the resterilized plate lid was replaced. The plate was connected to the Bioflux^™^ system, and the following sequence followed: 2 s at a shear of 2 dyne cm^−2^ to seed the microfluidic tube, 30 min no flow for cell adherence, 14 h at a shear of 0.5 dyne cm^−2^ and 1 h at a shear of 0.25 dyne cm^−2^. The selected shear conditions were chosen to maintain continuous nutrient flow over the duration of the experiment while approximating the low-shear environment associated with mucus accumulation and impaired clearance in CF airways. These values fall within the range reported in a previous Bioflux-based study of *P. aeruginosa* biofilm formation under physiologically relevant flow conditions [17]. Following the completion of the Bioflux^™^ run, the plate was disconnected, the lid was removed and imaging was proceeded with using the confocal microscope (Zeiss) as described below.

### Reverse transcription quantitative real-time PCR

The SCW was prepared as described above with either PAO1L or PAO1L Δ*aaaA* and was incubated at 37 °C for 16–40 h. Following incubation, the SCW plug was tipped into a 1.5-ml Eppendorf containing 400 µl RNA-Protect^™^ (QIAGEN), vortexed for 10 s and then incubated at room temperature for 5 min, as per the manufacturer’s instructions. Tubes were spun at 5,000 ***g*** per 10 min, the supernatant was gently removed by pouring and then, the inverted tube was dabbed gently on a paper towel for 5 s to dry and immediately frozen at −80 °C. For RNA extraction, the RNeasy mini extraction kit (QIAGEN) was used following protocol 5 for mechanical and enzymatic lysis according to the manufacturer’s instructions (QIAGEN, 2020 pg. 34). Previously frozen RNAprotect^®^ pellets were thawed on ice, and each was resuspended in 100 µl TE buffer (30 mM Tris-HCl, 1 mM EDTA, pH 8.0) containing 15 mg ml^−1^ lysozyme. Ten microlitres of proteinase K was added, and the mixture was incubated at 22 °C, 200 r.p.m. for 30 min. Following the addition of 700 µl buffer RLT, the mixture was then transferred to UV-sterilized 2 ml FastPrep^™^ safe-lock tubes containing 0.6 g lysis beads (150–600 µm diameter) and homogenized with two 30 s cycles at 6.0 m s^−1^ using the FastPrep-24 5G (MP Biomedicals^™^). Following lysis, tubes were centrifuged at maximum speed for 1 min, supernatant added to 590 µl 80% ethanol and mixed well. RNA was then purified using the RNeasy mini spin column kit according to protocol 7 (QIAGEN, 2020 pg. 40) and eluted in 10 µl nuclease-free water. The Turbo DNA free kit (Invitrogen) was used to remove genomic DNA according to the manufacturer’s protocol for rigorous DNase treatment, and a NanoDrop ND-1000 spectrophotometer (Nanodrop Technologies) was used to quantify RNA and check 260/280 for signs of impurities.

Using the Promega GoScript^™^ RT kit with random primers, cDNA was synthesized according to the manufacturer’s instructions. To do this, 200 ng of DNA-free RNA was added to each reaction. RT negative controls were also used, where no RT enzyme was added.

For reverse transcription quantitative PCR (qPCR), primer-specific master mixes for a final well volume of 25 µl were prepared. All reagents and consumables were sourced from Applied Biosystems. The master mix contained 0.625 µl each of forward and reverse primer (final concentration 250 nM), 12.5 µl 2X PowerSYBR Master mix and 10.25 µl NF H_2_O and was then added to a MicroAmp™ Optical 96-Well Reaction Plate. Then, 0.5 µl of cDNA (50 µg µl^−1^) was added to the respective wells, and the plate was sealed with a MicroAmp^™^ Optical 69 Adhesive Film. A *rpoD* reverse transcription-negative control was added for each template sample to check for the absence of gDNA. All reactions were run on an ABI7500 Fast qPCR machine within the same calibration timeframe. Thermocycling included an initial 10 min denaturation step at 95 °C, followed by 40 cycles of 15 s at 95 °C and 1 min at 60 °C, followed by determination of a melt curve. ΔΔCt and relative quantities were calculated using the ABI7500 software, using *rpoD* and *rpoS* as endogenous controls.

### Growth assay

Overnight cultures of *P. aeruginosa* were diluted into fresh LB media at an OD_600nm_ of 0.01, and 200 µl was added in triplicate for each strain into a 96-well plate. Plates were incubated at 37 °C in a TECAN Sparks, and OD_600nm_ (growth) was monitored every 30 min for 24 h.

### Live/Dead microscopy of the SCW model

Two hundred microlitres of SCW was prepared and inoculated into a µ-Slide 8-well high glass-bottom chamber and incubated in a humidity box for 24 h at 37 °C. Biological triplicates were inoculated with either PAO1L or PAO1L Δ*aaaA*, leaving two wells as non-infected controls. After 24 h, 50 µl SYTO-9 (25 µM) and 50 µLl PI (1 mg ml^−1^) were added to each well and incubated in the dark at 37 °C for 1 h. Each well was imaged using a Zeiss LSM 700 confocal microscope, at magnification 5×, 20× and 63×, using channels for SYTO-9 (laser 488 nm) and PI (laser 555 nm). Files were exported as .lsm, and Fiji was used to split the channels and calculate the max intensity from each Z stack. The % live to dead intensity was calculated using the following equation:


$$\%Live=\frac{SYTOintensity}{SYTOintensity+PIintensity}x100$$

% Dead=100 - % Live

Fiji was also used to add scale bars to images.

### Statistics

Data were collected and analysed using Microsoft Excel (2018 or later) and GraphPad Prism 9. GraphPad was used to conduct statistical analysis and create graphs. All data sets were first tested for normality using the Shapiro–Wilk normality test. When comparing between two groups of data, an unpaired t-test was used if normality was met, and a Mann–Whitney test was used, where the data were not normally distributed. When comparing more than two groups of data, if normality was not met, a Kruskal–Wallis test with multiple comparisons was used. If the data were normally distributed and equal standard deviations were assumed, a two-way ANOVA with multiple comparisons was conducted. Where equal standard deviations were not seen, a Brown–Forsythe and Welch’s ANOVA with Dunnett T3 multiple comparisons was conducted. Specific groups compared are stated in the relevant figure legends.

## Results

### AaaA is highly conserved across *P. aeruginosa* strains

To investigate the conservation of AaaA in *P. aeruginosa*, the *aaaA* gene was compared across numerous *P. aeruginosa* genomes using the online tool PanX and Pseudomonas.com databases.

**Fig. 1.**
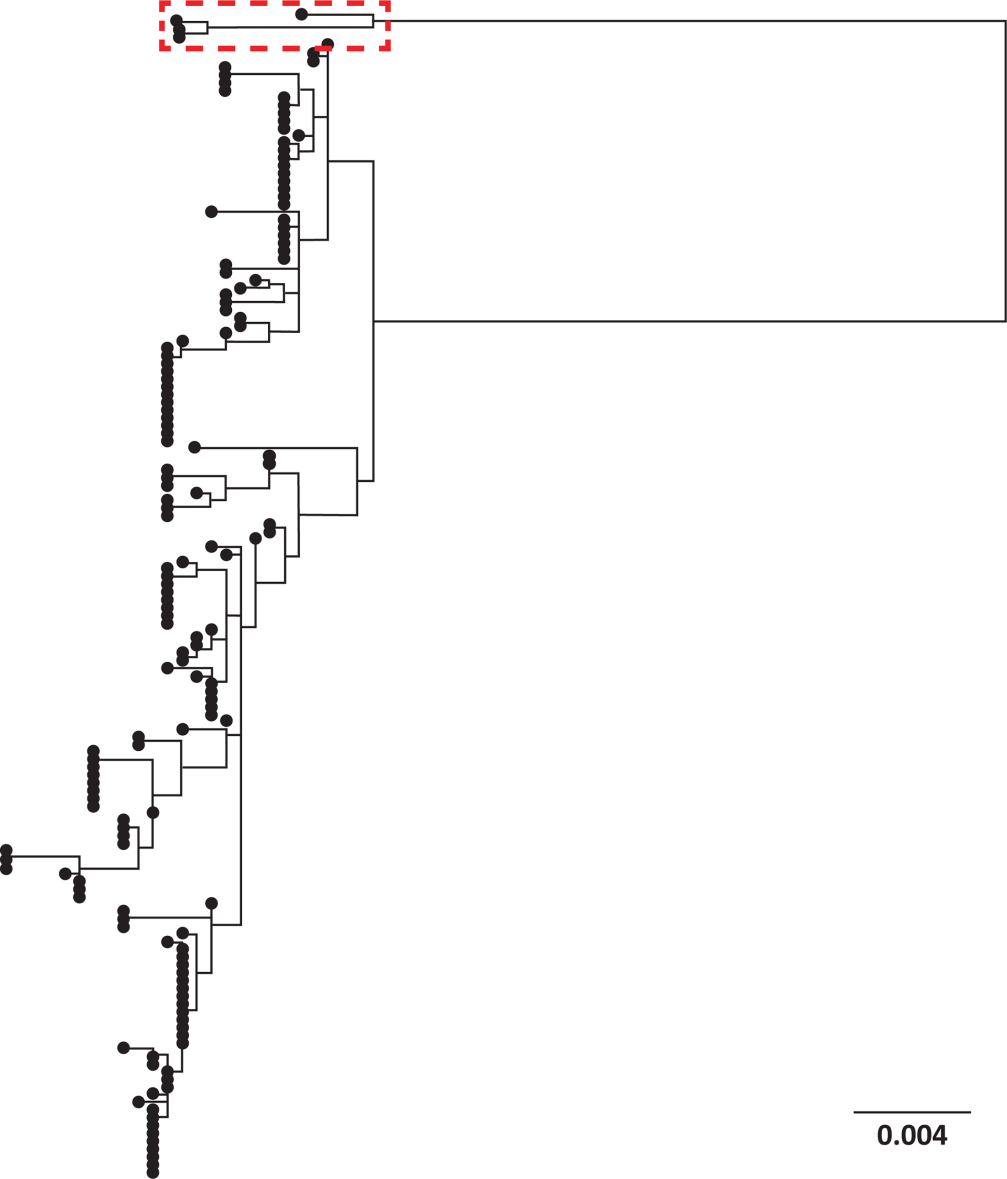
Tree of relatedness for AaaA generated using the PanX database of 153 *P. aeruginosa* genomes. Amino acid sequence identity is >99% for all but four isolates, which form a distinct clade (dashed red box). The horizontal scale represents 0.004 amino acid substitutions per site*.*

PanX allowed all genes from 153 *P*. *aeruginosa* genomes to be searched and aligned to the PAO1 genome. Within the 153 genomes searched, AaaA had a >99% protein sequence identity in all except four strains, which form a distinct clade (Fig. 1).

**Fig. 2.**
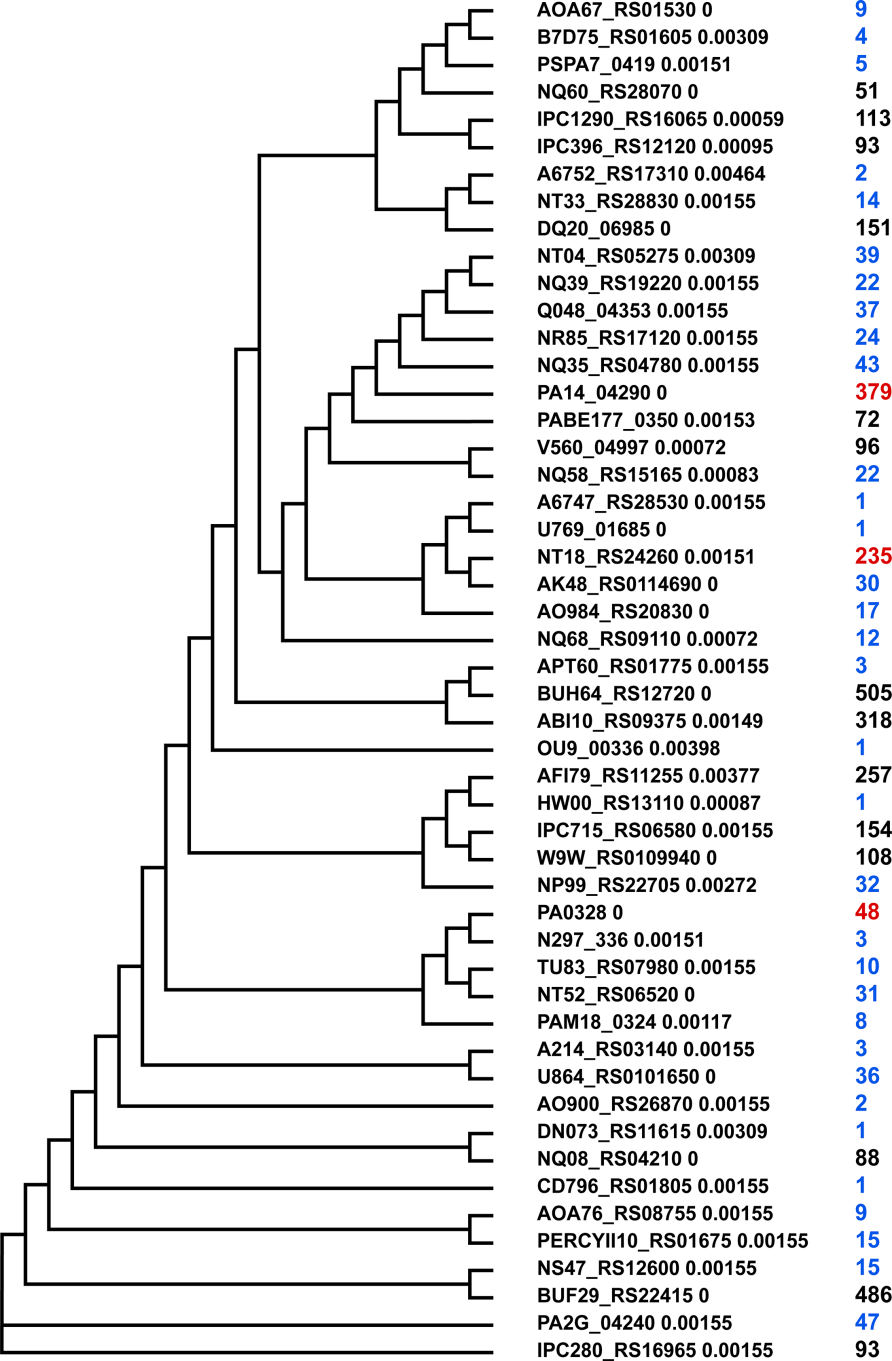
Cladogram showing representatives of all the unique complete sequences for AaaA in *P. aeruginosa* in the Pseudomonas.com database. Numbers immediately following the sequence ID indicate the evolutionary distance between the sequence and its diverging node. Numbers aligned to the right of the figure indicate the frequency of occurrence of each sequence, out of 3,748 genomes. Those that occur more and less commonly than PAO1 (PA0328) are shown in black and blue, respectively. The sequence for AaaA found in PAO1 and PA14 is shown in red, as well as N18_RS24260, which formed the distinct clade in Fig. 1.

This conservation was further explored using a larger genome database, Pseudomonas.com, which contained 4,669 genomes. A diamond blastp search was conducted to search stringently for missense variations. Of the 4,669 genomes searched, complete sequences (coverage 100%, identity 95%) of AaaA were present in 3,748 of the genomes. Within this group of genomes, there were only 50 unique sequences of an intact *aaaA* gene (Fig. 2). PROVEAN software was used to determine the likelihood of any of the mismatches identified in the 50 unique sequences to be deleterious on *aaaA* (Table S1, available in the online Supplementary Material). Most notably, there were no amino acid mismatches in the five residues known to contribute to the active site or AaaA surface expression in the entire population (H100, D102, D115, E147 and E148) [13]. Together, this highlights the extent of conservation of the AaaA structure and implies its role is important to the bacterium.

### AaaA activity assay shows a range of activity across clinical isolates.

To gain a realistic representation of the function of AaaA in chronic wounds, clinical isolates derived from patients were used and characterized. A range of *P. aeruginosa* clinical isolates were sampled from patients at Nottingham University Hospitals. All isolates originated from chronic diabetic ulcers with a mix of tissue and bone samples from both short-term, defined by the presence of *P. aeruginosa* in the wound for less than 6 months, and long-term infection (Table 5). The range of sampling methods and chronic wound duration allows the study of AaaA at different time points along the longitudinal patient history, allowing a better picture to be drawn about its role in chronic infection.

**Table 5.** Infection information for each clinical isolate strain

Patient	Wound location	OM	Duration (months)	LT
**CW2**	Big toe	Yes	3	No
**CW3**	Calcaneal ulcer	Yes	5	No
**CW4**	Calcaneal ulcer	Yes	25	Yes
**CW5**	Calcaneal ulcer	Yes	26	Yes
**CW6**	Heel ulcer	No	36	Yes
**CW7**	Leg ulcer	No	3	No

OM, osteomyelitis at time of sampling. Duration: time since the first recorded identification of *P. aeruginosa* or *S. aureus* in the wound. LT, long-term infection defined by the presence of *P. aeruginosa* for more than 6 months.

**Fig. 3.**
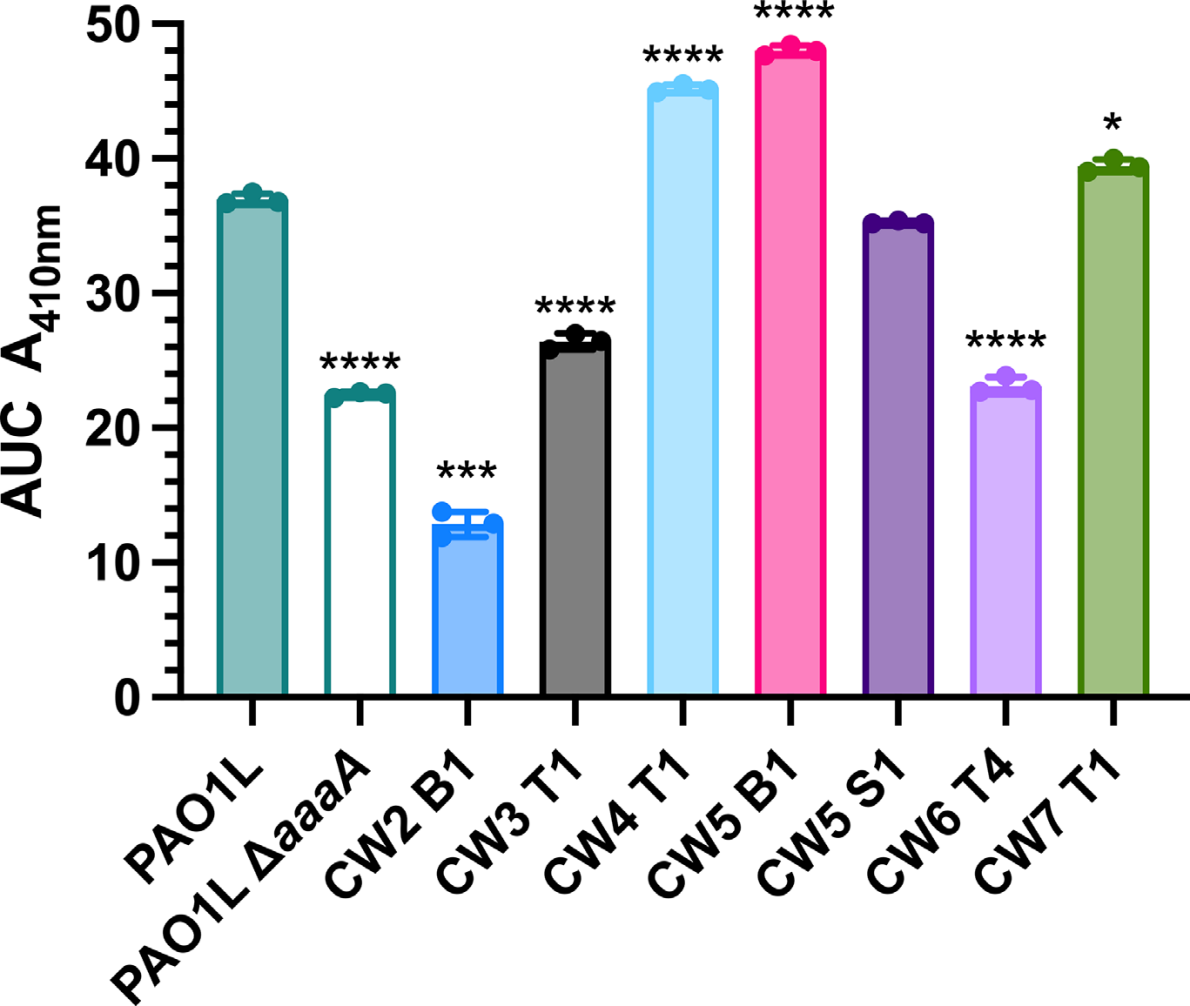
AaaA activity assay showed varying levels of intrinsic AaaA activity in *P. aeruginosa* skin wound clinical isolates. l-Arginine-p-nitroanilide cleavage by AaaA in PAO1L, PAO1L ΔaaaA and seven chronic wound isolates (CW2–7, Table 5) was measured over 24 h. Strains were grown in LB to mid-exponential phase (OD_600nm_ 0.5–0.8) before the AaaA assay was conducted. Absorbance was monitored at 410 nm. Significant differences were determined by Brown–Forsythe and Welch’s ANOVA (F* (DFn, DFd) 3094 (11.00, 11.61) *P*<0.0001), with Dunnett’s T3 multiple comparisons comparing each group to PAO1L wild-type. *P<0.05, ***P<0.001, ****P<0.0001. *n*=3 repeats, mean±sd bars*.*

A whole-cell activity assay was conducted on all the clinical isolates to determine intrinsic levels of AaaA activity. A range of AaaA activity was seen in the isolates (Fig. 3). Notably, both CW2 B1 and CW4 T1 were co-isolated alongside a methicillin-sensitive *S. aureus*, which provides the opportunity to study AaaA activity in a polymicrobial environment [18].

CW2 B1 had the lowest levels of AaaA activity, with CW3 T1 and CW6 T4 also having low levels comparable to PAO1L Δ*aaaA*. CW4 T1, CW5 B1 and CW7 T1 all have AaaA activity significantly higher than PAO1L. From the AaaA activity assay results, CW2 B1, CW4 T1 and CW5 B1 were selected as clinical backgrounds in which to study the function of AaaA further. As seen in Table 5, this provides a range of samples from different chronic wound types, which have been infected with *P. aeruginosa* for different timeframes. It also provides isolates with both a ‘high’ and ‘low’ level of intrinsic AaaA activity for comparison.

To create a more clinically relevant background in which to study AaaA, an *aaaA* knockout mutant was constructed in each of the selected *P. aeruginosa* chronic wound isolates CW2 B1, CW4 T1 and CW5 B1, alongside PAO1L, as described by Hmelo *et al.* [16] using the pEX18Gm:Δ*aaaA* plasmid. Successful knockout of *aaaA* was confirmed using WGS. Genetic complementation strains for the Δ*aaaA* clinical strains were constructed using the chromosomally stably inserted miniCTX-*aaaA* plasmid (Table 2). Phenotypic confirmation of AaaA knockout and complementation was then completed using a whole-cell AaaA activity assay (Fig. S3).

### Optimization of the SCW model to study AaaA activity

**Fig. 4.**
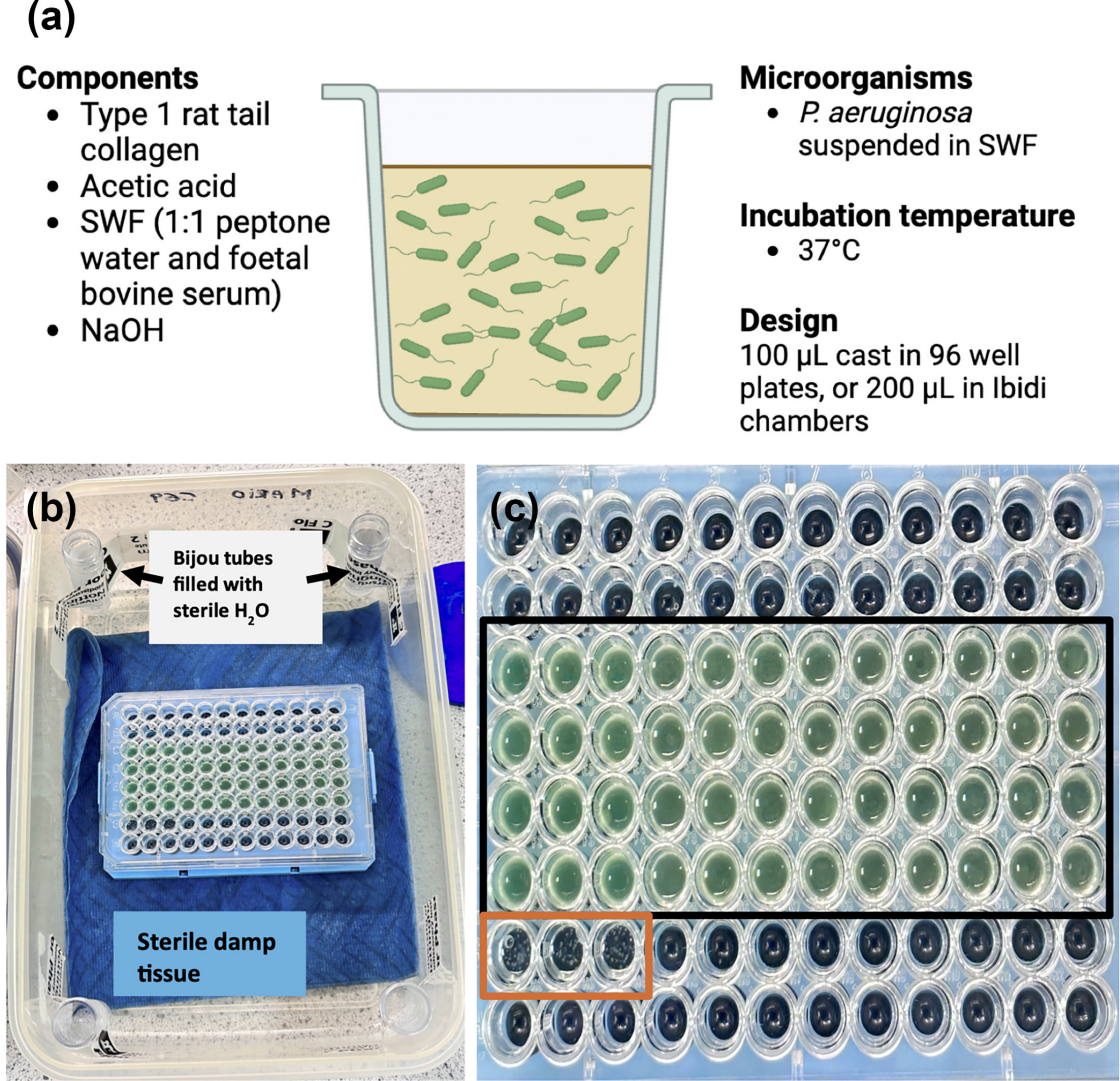
The SCW model used in this study. The SCW was grown in a 96-well plate incubated statically at 37 °C for 16, 24 or 48 h inside a sterilized humidity-tight box (shown in panel a). The green pigmented compounds pyocyanin and pyoverdine are produced by *P. aeruginosa* and are visible in the infected wells (within the black box) but not in the non-infected controls (wells G1–G3 in the orange box) in panels (b/c). The remaining wells were not used*.*

To assess *aaaA* expression and AaaA activity under conditions where a phenotype is more likely to be observed, a SCW model was employed, as AaaA has previously been associated with chronic infection and shows limited phenotype under planktonic conditions [13, 19]. The original model [20] has been further developed to mimic the matrix and chemical components found in a chronic wound while being minimalistic and reproducible to construct in a laboratory (Fig. 4). Specifically, type I rat tail collagen was combined with SWF, comprising FBS and peptone, to generate a matrix and wound-like exudate. In addition, collagenase-mediated degradation was optimized to enable complete homogenization of the biofilm for downstream AaaA activity assays and c.f.u. quantification. The SCW model is compatible with both 96-well plates for high-throughput screening and 8-well chambers for imaging applications.

### *aaaA* is most highly transcribed at 16 h in the SCW model

**Fig. 5.**
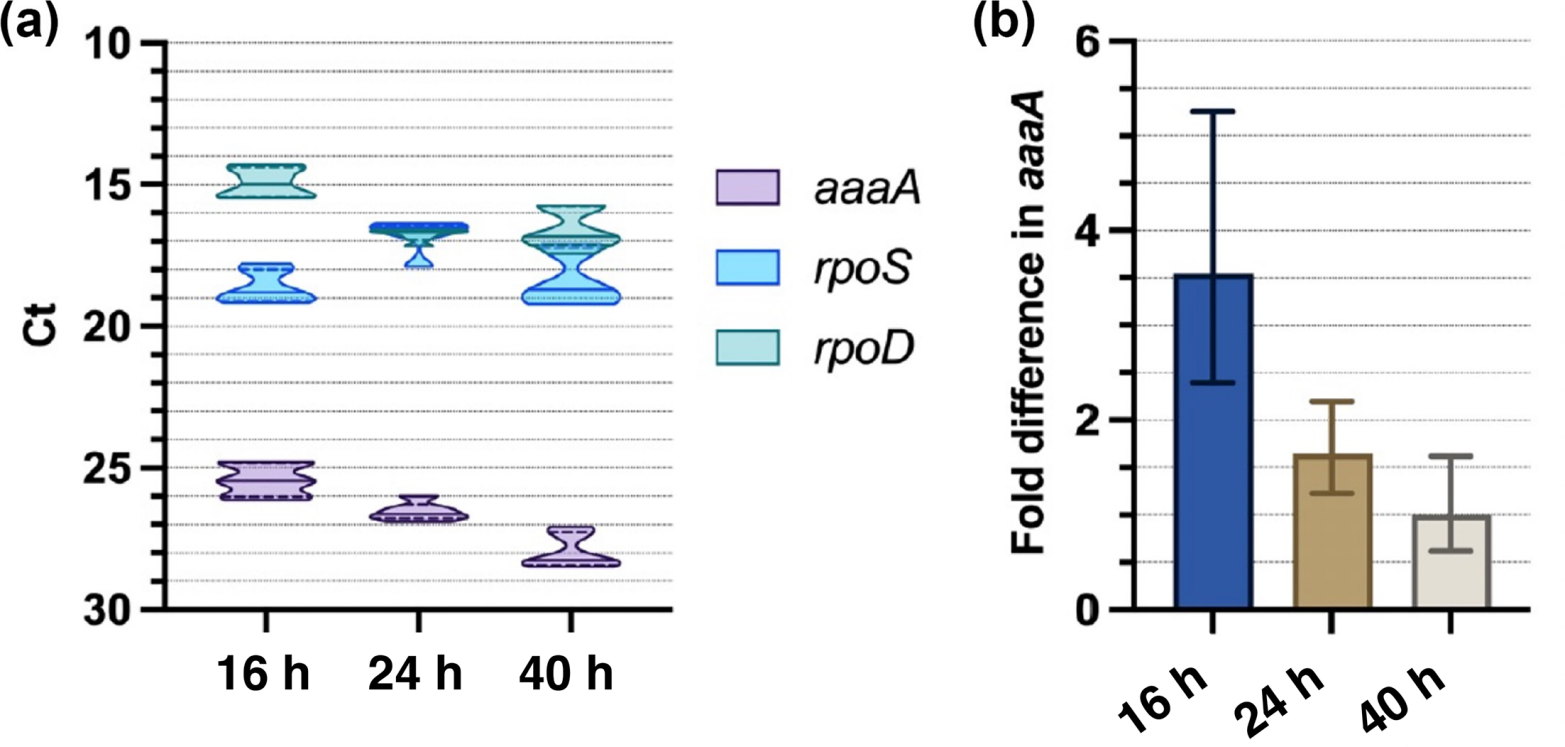
Reverse transcription qPCR of SCW for relative quantification of *aaaA* over time shows that *aaaA* is most highly transcribed at 16 h. Panel (a) is a truncated violin plot of the Cts for each sample (median as line, quartiles as dashed lines, *n*=6, 2 technical and 3 biological). *rpoS* and *rpoD* were used as exogenous controls to calculate the relative quantity of *aaaA* at each time point, shown in panel (b) as fold difference (40 h used as an arbitrary reference with fold change set to 1). ΔΔCt for each time point was –1.8277, –0.7163 and 0 for 16 h, 24 h and 40 h, respectively*.*

The relative mRNA levels of *aaaA* were quantified in PAO1L samples after growth in the SCW model for 16, 24 and 40 h, using *rpoD* and *rpoS* as endogenous controls. Transcripts for *aaaA* could be detected at all timepoints tested, with the highest relative levels seen after 16 h in the SCW model (Fig. 5).

### AaaA activity is higher in the SCW model compared to planktonic LB and SWF

**Fig. 6.**
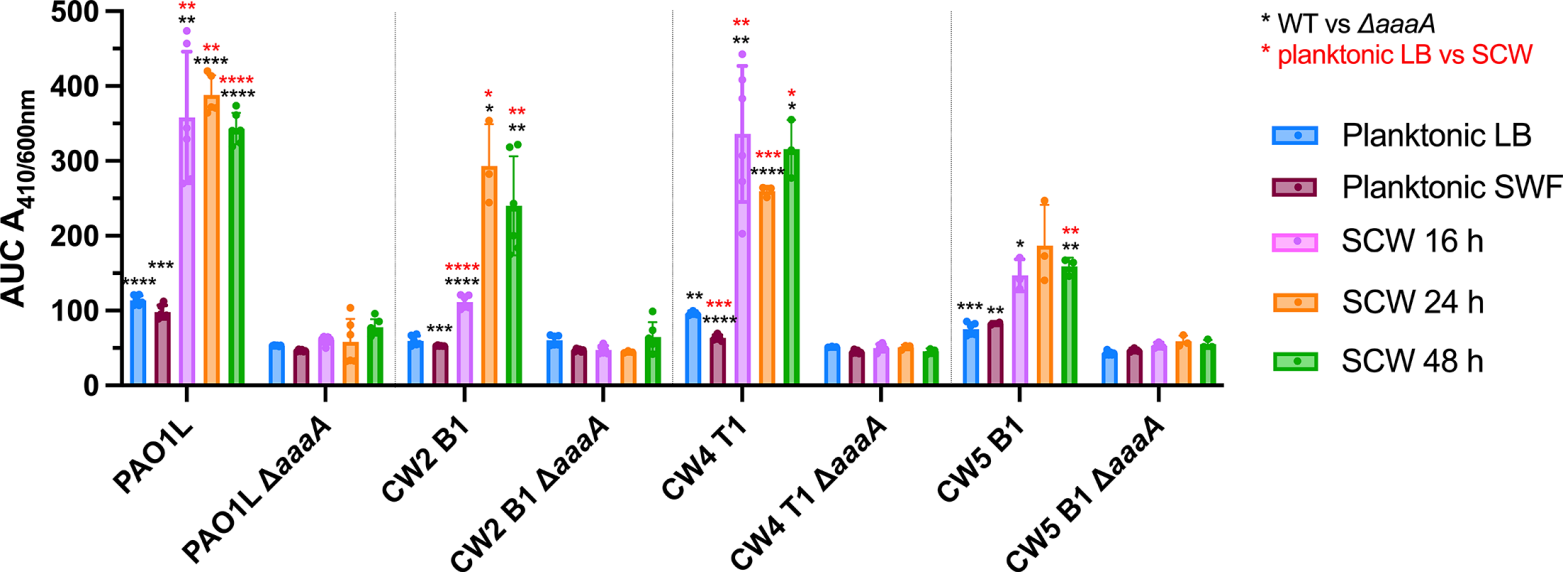
AaaA activity in different *P. aeruginosa* strains in planktonic and SCW contexts. Cells were grown either planktonically in LB or SWF to mid-late exponential phase (OD_600nm_ 0.6–0.9) or in the SCW for 16, 24 or 48 h, and 100 µl was added to the activity assay (*n*=3 biological repeats). Significance was determined by Brown–Forsythe and Welch’s ANOVA (F* (DFn, DFd) 125.6 (8.00, 22.07) *P*<0.0001), with Dunnett’s T3 multiple comparisons comparing between WT and ΔaaaA in black and between growth conditions in red. *P<0.05, **P<0.01, ***P<0.001, ****P<0.0001. Mean±sd bars shown*.*

To determine whether AaaA activity is preferentially associated with biofilm growth rather than planktonic conditions, AaaA activity in PAO1L, CW2 B1, CW4 T1 and CW5 B1 after growth in the SCW for 16, 24 or 48 h was compared to planktonic AaaA activity after growth in LB and SWF. Growth of all strains was comparable across all conditions tested, as the strains were suspended in MMP for the AaaA activity assay. The derived *aaaA*-deficient mutants were included as negative controls for comparison. As seen in Fig. 6, while comparable levels of AaaA activity were measured in planktonic LB and SWF cultures, higher levels of AaaA activity are seen in the parental strains when grown in the SCW for 16 h or more.

All strains showed significant increases in AaaA activity when comparing planktonic LB to SCW, with this significance being consistent for the different incubation times of the model for all strains except CW5 B1. Although this did not reach statistical significance for CW5 B1, a consistent trend towards an increase in AaaA activity at 16 and 24 h in SCW compared to planktonic LB was evident. The highest levels of AaaA activity, except CW2 B1 and to a certain degree CW5 B1, were at the 16 h timepoint in SCW model, which is in agreement with the analysis of transcription in PAO1L (Fig. 5). The variance between clinical isolates, e.g. the exception seen in CW2 B1, is not surprising given the selective pressures that they will have been exposed to and the likely adaptation of regulatory factors.

As expected, a statistically significant loss in AaaA activity was measured in the Δ*aaaA* mutants derived from PAO1L, CW2 B1, CW4 T1 and CW5 B1. Although statistical significance was not observed for CW5 B1 after 24 h in the SCW, a trend towards reduced AaaA activity in the Δ*aaaA* mutant, consistent with the other strains, was evident.

**Fig. 7.**
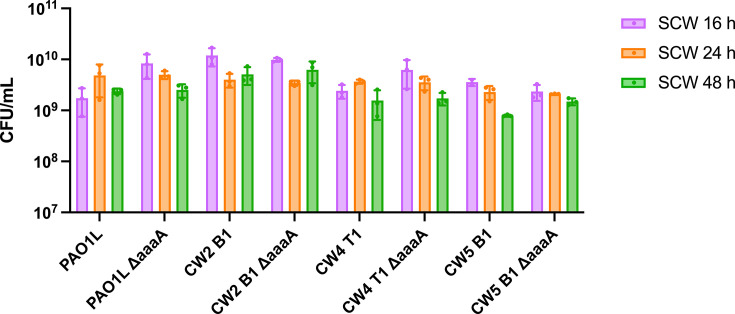
c.f.u in different *P. aeruginosa* strains in SCW contexts. Cells were grown either in the SCW for 16, 24 or 48 h. Samples were homogenized with 100 µl of 500 µg ml^−1^ of collagenase, and then, 20 µl was serially diluted in 180 µl PBS. Dilutions of 10^−3^ to 10^−8^ were plated on LB using the Miles and Misra method. No significance seen (Brown–Forsythe and Welch’s ANOVA (F* (DFn, DFd) 8.693 (7.00, 6.275) *P*=0.0076), with Dunnett’s T3 multiple comparisons comparing between WT and ΔaaaA and between growth conditions). *n*=3 biological repeats mean±sd bars plotted*.*

There was also no significance in c.f.u. between WT and Δ*aaaA* in the SCW model (Fig. 7). This suggests that AaaA does not confer a measurable difference in overall cell numbers under these conditions. This is further supported by comparable growth kinetics observed between WT and Δ*aaaA* strains in planktonic LB culture (Fig. S4). There was also no significant difference in viability between each of the SCW timepoints, which implies that the current SCW model could be used for longer time periods, as the stabilization of cell number points to robust nutrient availability.

### AaaA plays a role in biofilm formation and morphology

**Fig. 8.**
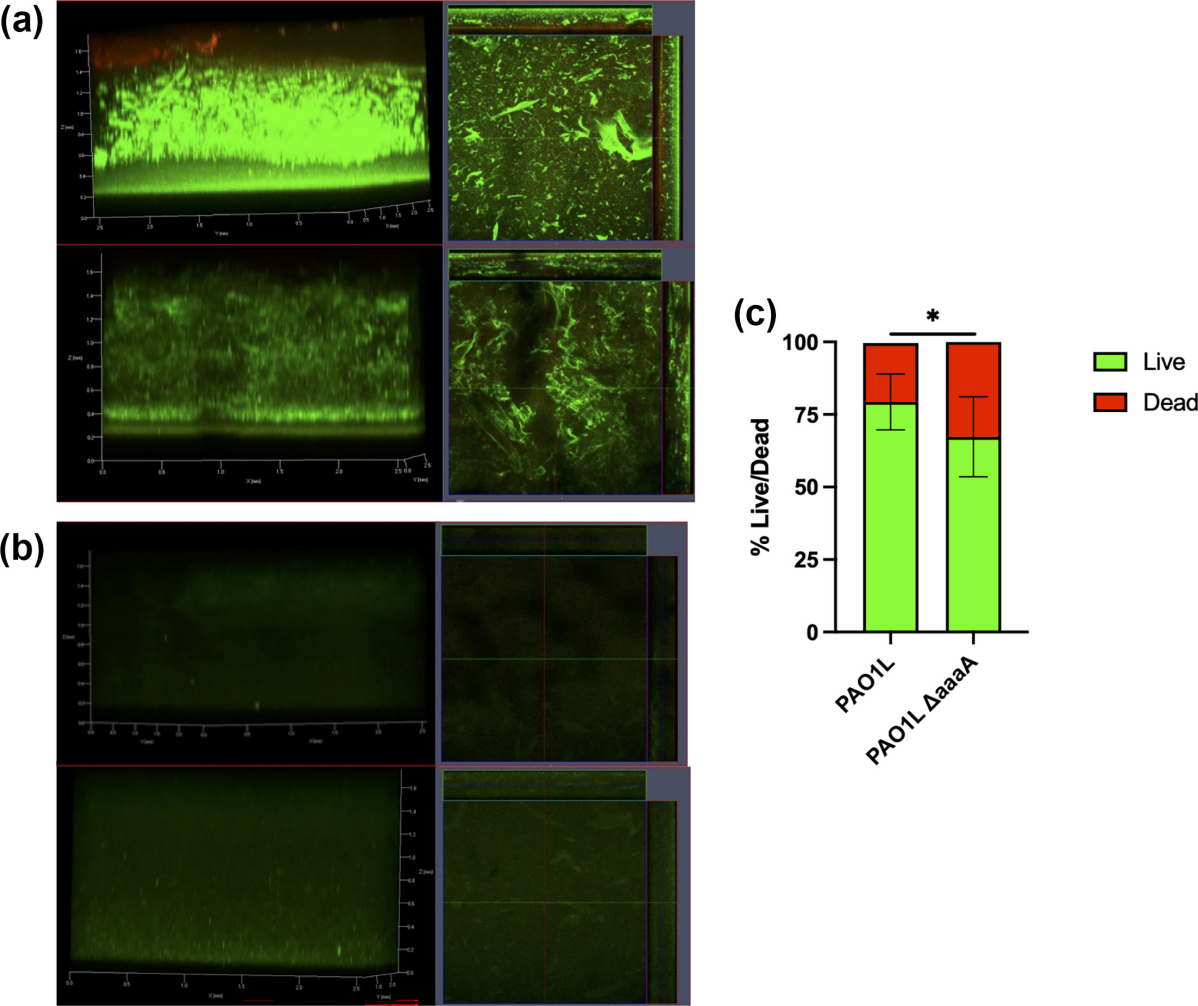
Live/dead staining shows AaaA plays a role in biofilm morphology in SCW. Panels (a) and (b) show 3D projections of 5× magnification z-stack images of 24 h SCW infected with either PAO1L WT (panel a) or PAO1L ΔaaaA mutant (panel b) on the left and orthogonal views on the right. Two representative sets of images are shown for each strain in the upper and lower rows of each panel. The percentage of live/dead cells is shown in panel (c). The mean (±sd) ratio of live/dead cells as a percentage was calculated from three biological repeats of each condition, imaging three points in each well. Percentages compared using Welch’s t-test (*n*=9, *P=0.0323).

Since AaaA is active in the SCW, and because the formation of biofilm communities is key to establishing chronic infections *in vivo*, the effect of AaaA on biofilm morphology was investigated in the SCW. Growth and morphology of biofilms formed by PAO1L WT and the derived Δ*aaaA* mutant in the SCW were compared using a live/dead staining involving SYTO-9 and PI. Confocal microscopy 5× magnification z-stacks of the stained biofilms revealed a difference in biofilm morphology and distribution of bacteria within the SCW (Fig. 8).

Biofilms produced by PAO1L have a denser distribution (Fig. 8a), with significantly more live cells (green) compared to biofilms produced by PAO1L Δ*aaaA* (Fig. 8b), as seen in Fig. 8(c). The WT biofilm is also produced at a deeper depth in the collagen matrix and spans a much larger area, as evidenced by the larger z-stacks in Fig. 8A, with PAO1L biofilms having a depth of around 1.4 mm. In contrast, PAO1L Δ*aaaA* biofilms produce a much sparser surface-level biofilm, with a depth of around 0.4 mm, that is closer to the top of the collagen matrix. This supports the theory that AaaA could be an important protein in the anaerobic pathways of *P. aeruginosa*, as biofilms are produced closer to the air interface in Δ*aaaA* mutants. The orthogonal view (on the right of Fig. 8) shows the difference in biofilms produced at equivalent depths. In the WT, there seem to be aggregates of live cells that are not present in the biofilms of the Δ*aaaA* mutant. This suggests that AaaA contributes to biofilm structure and organisation within a chronic wound environment. Reduced thickness biofilms were also seen in a cystic fibrosis *aaaA* mutant, LESB65 Δ*aaaA,* when grown in a microfluidic system in ASM (Bioflux^™^) (Fig. S5). The consistent effect that the absence of *aaaA* has on biofilm formation, which is seen in different strain backgrounds and biofilm models, supports the theory that AaaA plays an important role in biofilm formation.

**Fig. 9.**
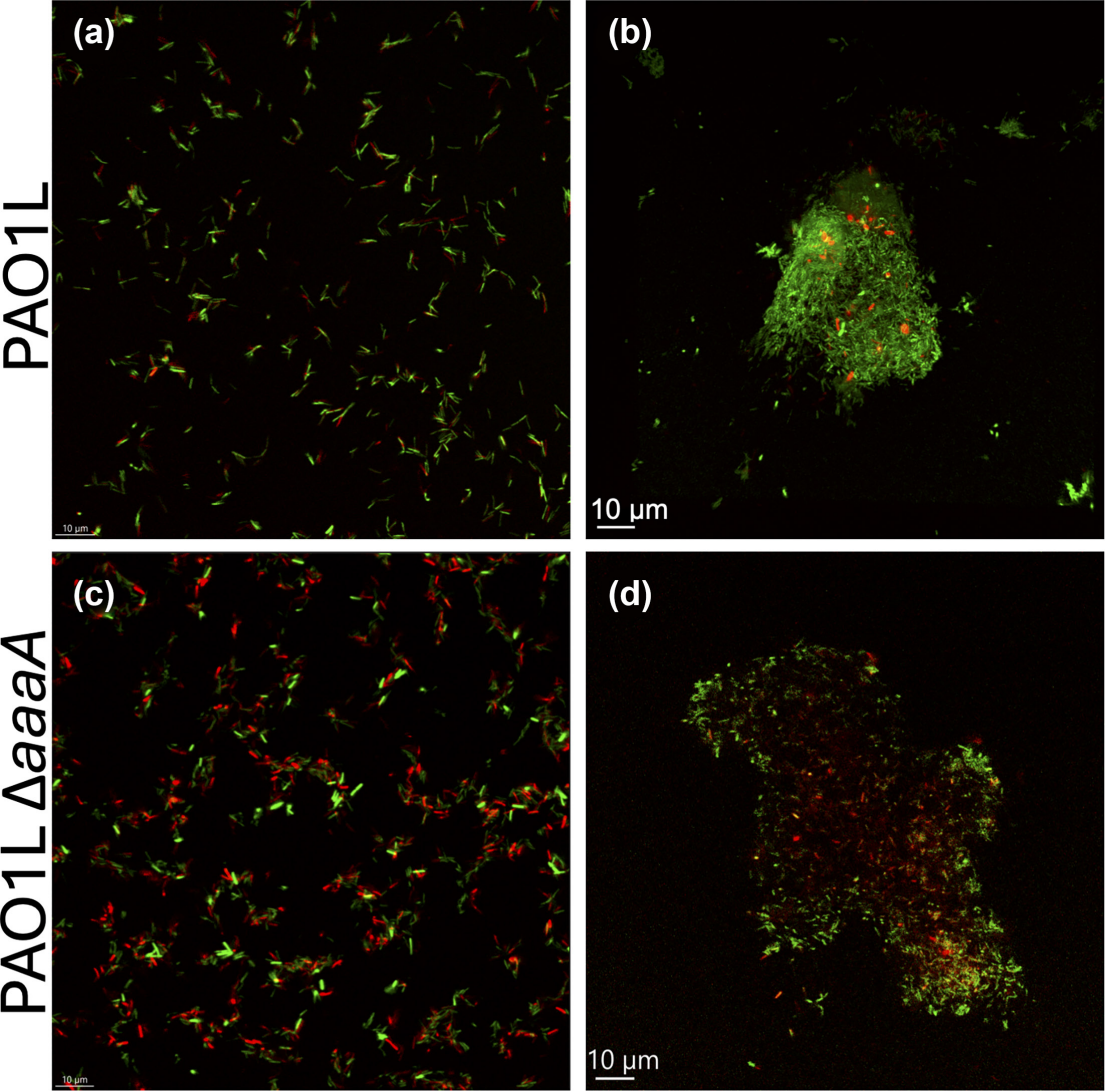
PAO1L WT (panel a+**b** ID=‘c6c7ac4c-3b06-4c8e-839f-d46d371b7af3’>) is more viable than PAO1L ΔaaaΑ (panel c+**d** ID=‘ece1a193-943d-478c-af56-cc3f07ab2ae0’>) in a 24-h SCW model. Live/dead staining was conducted after 24-h incubation in the SCW model, and biofilms were visualized at 63× magnification with confocal microscopy. (a) and (c) show the bottom of the biofilms, and (b) and (d) display aggregates of bacteria found within the collagen matrix*.*

Images were also taken at a higher magnification in the SCW model to visualize the individual bacterial cells and aggregates formed (Fig. 9). At 63× magnification, it was possible to see both individual *Pseudomonas* cells and aggregates of cells at the bottom of each well. Although bacterial aggregates were present in both WT and Δ*aaaA* mutant biofilms, the bacterial aggregates formed by the WT incorporated a higher level of live (green) cells, whereas aggregates formed by the deletion mutant contained many more dead (red) cells, especially at the centre of the mass. There were evidently more dead individual cells at the bottom of the Δ*aaaA* plug compared to the WT. This suggests that AaaA may influence cell viability within biofilm structures in the SCW model. This also demonstrates that AaaA provides a survival advantage within the SCW model.

## Discussion

### AaaA is produced and active in the SCW model

Luckett *et al.* [13] showed that the PAO1 Δ*aaaA* mutant is deficient in long-term survival in a mouse chronic wound infection model [13]. This led to the hypothesis that AaaA is important in a chronic wound context. This is further supported by RNA-seq data showing *aaaA* is upregulated by a 3.21-fold change in chronic wounds and 3.92-fold change in burns when compared to MOPS-succinate media [21]. Here, AaaA activity was examined in the SCW model in PAO1L and a range of chronic wound clinical isolates from both long-term and short-term infections. AaaA activity was higher in all strains in the SCW model compared to planktonic LB and SWF growth (Fig. 6). Interestingly, CW2 B1 exhibited intrinsically low levels of AaaA planktonically, but significantly higher levels after growth in the SCW model. In contrast, this trend was not observed in the other clinical isolates, which were derived from longer-term infections (25 and 26 months for CW4 T1 and CW5 B1, respectively, Table 5), whereas CW2 B1 was isolated from a relatively early-stage infection (3 months). It is also notable that AaaA activity in the CW2 B1 WT strain was lower than that observed in the PAO1L Δ*aaaA* mutant (Fig. 3). This highlights that absolute activity levels vary between strain backgrounds and suggests that the measured activity may be influenced by factors beyond AaaA alone, such as background protease activity or strain-specific differences in gene expression. Therefore, comparisons of AaaA function are most appropriately made within the same genetic background, rather than between different isolates. Studies have shown that bacterial cells modify gene expression in long-term infections due to changes in the environment and different nutritional needs [22]. In line with this, AaaA production may vary over the course of infection, for example, increasing during early biofilm establishment and being modulated at later stages. This could explain why clinical isolates from shorter-term infections, such as CW2 B1, exhibit increased AaaA activity in the SCW model but lower activity during planktonic growth in nutrient-rich conditions (Fig. 6). However, whether AaaA expression imposes a fitness burden was not directly assessed in this study.

No significant difference in CFUs was seen at any of the timepoints tested between WT and Δ*aaaA* mutant strains, potentially countering the hypothesis that AaaA could provide a survival advantage in chronic infection. AaaA was previously shown to be important in chronic infection after 8 days of infection [13]. In the SCW model, the longest timepoint tested is 48 h, and so a longer incubation time may be needed to see a survival advantage provided by AaaA. Follow-on studies over longer time periods, monitoring chronicity with a biomarker of the chronic-acute transition such as SicX [23], might reveal a significance between WT and Δ*aaaA.* However, as nutrients are not replenished in this model, there is a limit to how long bacterial growth can be monitored.

Overall, these findings show that AaaA activity varies between strains but is generally increased in disease-relevant backgrounds and models when compared to planktonic LB media conditions. This implies that the SCW model is an appropriate model for studying AaaA. Despite the high level of sequence conservation observed for AaaA across *P. aeruginosa* genomes (Figs 1 and 2, Table 2), AaaA activity was found to vary between strains in this study. This suggests that differences in AaaA function are likely driven by regulatory mechanisms rather than structural variation in the protein itself. Such variability may reflect adaptation to different infection environments or stages, where modulation of *aaaA* expression could contribute to strain-specific phenotypes in biofilm formation and chronic infection. Now that a strong and reliable phenotype for AaaA activity has been identified, further work to characterize both PAO1L and the chronic wound clinical isolates can be conducted. This has the potential to reveal more information about both the regulatory control of *aaaA* and the importance and function of AaaA. This study was conducted in aerobic conditions. However, due to the microaerobic and anaerobic nature of chronic wound infections, it would be interesting to explore the function of AaaA in the SCW under anaerobic conditions, to more closely mimic the chronic infection environment [24].

### AaaA influences biofilm morphology and organization

Microscopy was used to determine the effect AaaA has on bacterial cell survival and biofilm formation in different models. Using the optimized chronic wound biofilm model, z-stack data from live/dead staining (Fig. 8) showed that AaaA plays a role in biofilm morphology, with larger, denser z-stacks seen in the WT strain compared to the Δ*aaaA* mutant. LESB65, a cystic fibrosis isolate, also produced thinner biofilms in an *aaaA* deletion mutant when grown in ASM in a Bioflux^™^ model (Fig. S5). These results concur with the findings in Fig. 8 and so are in line with AaaA playing a role in dispersion and bacterial survival. The more diffuse biofilms produced by the Δ*aaaA* mutant could be due to the anaerobic environment present within the biofilm and bacterial aggregates. Within the anaerobic environment, PAO1L would have to rely on AaaA to cleave the l-arginine vital for the arginine fermentation pathway, an anaerobic cycle of energy production used when nitrate sources are scarce [9]. However, as the mutant lacks *aaaA*, this anaerobic pathway cannot be utilized, forcing the bacteria to either revert to aerobic methods of energy production or use pyruvate fermentation, and so smaller bacterial aggregates and thinner biofilms are formed. This is evident in the 63× magnification images where bacterial aggregates of the same size show more cell death at the centre of the Δ*aaaA* mutant biofilms (Fig. 9). However, complementation studies would be required to definitively confirm this theory.

Other biofilm stains, such as eDNA or exopolysaccharide stains, could also be used to further quantify the biofilms formed by each strain. However, appropriate stains will have to be selected as both collagen and *P. aeruginosa* exhibit autofluorescence. Collagen has an excitation wavelength of 488 nm and *P. aeruginosa* excites at 274 nm, meaning that stains with excitation wavelengths around these values will have to be avoided [25, 26]. Crystal violet staining for the quantification of biofilm biomass is also not an option, as this dye binds to ribose-type molecules, which are also present in collagen [27]. As the SCW is so thick, washing the unbound crystal violet from the biofilm would also present an issue. Because of this, other methods of biofilm staining and quantification need to be optimized before these studies can be undertaken. Microscopy should also be conducted on the chronic wound clinical isolates after growth in the SCW model. This could determine if the pronounced differences in biofilm formation between WT and Δ*aaaA* also occur in clinical strains. It would also be interesting to compare biofilm morphology in a strain with intrinsically low levels of AaaA activity, CW2 B1, to strains with high AaaA activity, CW4 T1 and CW5 B1.

Although a significant difference was seen in the amount of live to dead cells in the WT compared to the Δ*aaaA* mutant after live/dead confocal microscopy, no significance was seen in the c.f.u. between the two strains. This could be due to the biofilm morphological differences between the two strains, where the Δ*aaaA* mutant forms more diffuse aggregates with increased localized cell death. It is also worth noting that confocal microscopy only takes a random snapshot of a few areas of the biofilm, whereas c.f.u. is conducted on the whole homogenized biofilm. Technical factors, such as propidium iodide staining of extracellular DNA and the presence of viable but non-culturable cells (VBNCs), may also contribute to this difference [28, 29]. As a result, further imaging with other stains and fluorescent proteins is needed to further quantify the differences seen in biofilm morphology between WT and Δ*aaaA* mutant strains. Other techniques, such as live/dead viability PCR, could also be used to bridge the gap between the confocal microscopy data and the CFUs [30]. This could also establish the presence of VBNCs and determine if there is a difference in cell viability between WT and Δ*aaaA* strains in both lab and clinical isolates.

In conclusion, this study demonstrated that AaaA activity depends on the particular clinical isolates biopsied from different chronic wounds. This activity could be dependent on the duration of infection, co-localization with other bacterial species, or could be a result of SNPs within the *aaaA* gene or elsewhere in the genome, e.g. within a regulatory element. Given that chronic wound infections are typically polymicrobial, interactions with co-infecting species such as *S. aureus* may influence AaaA activity, for example, through competition for nutrients, modulation of local environmental conditions or interspecies signalling. Such interactions could contribute to the variability in AaaA activity observed between clinical isolates. Through AaaA activity assays, it was shown that AaaA is more highly produced and active in the SCW than in planktonic LB culture, further supporting the theory that AaaA plays an important role in chronic infection. Moreover, novel evidence that AaaA has a role in biofilm formation within the SCW was obtained, since the Δ*aaaA* mutant biofilms were less dense with fewer aggregates and live cells. Taken together, it can be concluded that the SCW model is a viable option for the study of AaaA, and AaaA plays an important role in both chronic infection and biofilm formation. In so doing, AaaA has served to guide and verify the relevance of the optimization of the SCW model itself in readiness for its application to separate investigations into the fundamental microbiology of chronic wound infections. Furthermore, given that AaaA is surface-exposed, conserved and active under disease-relevant conditions, these findings support its potential as a therapeutic or vaccine target. Its involvement in biofilm structure suggests that targeting AaaA could interfere with chronic infection persistence, although further studies will be required to fully evaluate its clinical applicability.

## Supplementary material

10.1099/mic.0.001719 Supplementary Material 1.

## Abbreviations

ASM: artificial sputum media
CF: cystic fibrosis
iNOS: inducible nitric oxide synthase
LB: lysogeny broth
MMP: minimal media P
NEB: New England Biolabs
NF: nuclease-free
PI: propidium iodide
PIA: *Pseudomonas* Isolation Agar
qPCR: quantitative PCR
SCW: synthetic chronic wound
SWF: synthetic wound fluid
VBNCs: viable but non-culturable cells
WGS: whole-genome sequencing
