# Supplementary material for: Altered distribution of chronicity-deficient Pseudomonas aeruginosa mutant guides optimization of synthetic chronic wound model
Source: Microbiology (Reading). 2026 Jul 28;172(7):001719. doi: 10.1099/mic.0.001719 (PMC13418428; doi:10.1099/mic.0.001719)
Supplement: Supplementary Material 1. [file mic-172-01719-s001.pdf]

Supplementary Material

Altered distribution of chronicity deficient *P. aeruginosa* mutant guides optimisation of synthetic chronic wound model.

Bethan Roberts[1], Claire S. Laxton[1,2], Daniella Spencer[1,3], Ida Thaarup[4], Stephan Heeb[1], Christopher Penfold[1], Kim R. Hardie[1]

Table S-1 – PROVEAN scores for all amino acid substitutions identified in aaaA in the Pseudomonas.com database. *Using PA0328 as the reference genome, deleterious substitutions were predicted using 228 fasta sequences and respective E-values , in 30 clusters. Variations that are either high in occurrence, or potentially deleterious are highlighted pink and yellow, respectively.*

Variant	PROVEAN score	Prediction (cutoff= -2.5)	Variant	PROVEAN score	Prediction (cutoff= -2.5)
M1V	-1.401	Neutral	T407I	-0.931	Neutral
G10S	0.113	Neutral	G410A	0.256	Neutral
L18S	0.237	Neutral	N441H	-0.290	Neutral
S54T	-0.800	Neutral	E446Q	-0.037	Neutral
K55E	1.250	Neutral (high occurrence)	S464P	-0.399	Neutral
Y65H	-4.255	Deleterious (15 occurrences)	S488P	-0.380	Neutral
A75T	0.850	Neutral	R505Q	-0.381	Neutral
F94V	0.754	Neutral	S506N	-0.267	Neutral
G98S	-2.219	Neutral	H516R	0.174	Neutral (high occurrence)
R128H	-1.132	Neutral	A558P	-0.247	Neutral
G132D	0.251	Neutral	V571I	-0.067	Neutral
A167T	-0.465	Neutral	D576N	-1.000	Neutral
L170Q	-3.936	Deleterious (266 occurrences)	D580E	-0.339	Neutral (moderate occurrence)
K183R	-0.548	Neutral	D581A	-0.433	Neutral
S194A	-0.223	Neutral	M582I	0.160	Neutral
N195S	-0.125	Neutral (high occurrence)	T585S	-0.550	Neutral
F217L	0.311	Neutral	S586N	-0.267	Neutral
F217S	1.228	Neutral	V592I	-0.080	Neutral
A228T	-0.821	Neutral	V594G	-0.262	Neutral
D243N	0.644	Neutral	A595T	-0.083	Neutral
A251V	-3.727	Deleterious (1 occurrence)	M597L	-0.000	Neutral
N267D	-1.978	Neutral	M597I	0.033	Neutral
A280T	0.349	Neutral	G599D	0.250	Neutral
T288N	0.686	Neutral	K602R	-0.489	Neutral
N289G	-2.069	Neutral	A607V	-0.433	Neutral
L291F	0.048	Neutral	L609I	0.008	Neutral
E297D	-1.008	Neutral	L609F	-0.563	Neutral
Q314L	3.197	Neutral (high occurrence)	A611T	-0.117	Neutral
N359S	0.105	Neutral	A611V	0.300	Neutral
G363A	-0.656	Neutral	I615V	0.033	Neutral
E368K	-0.611	Neutral	V621I	0.008	Neutral
N386D	0.239	Neutral (moderate occurrence)	G638S	-0.433	Neutral
G393S	-0.156	Neutral	N644S	-0.267	Neutral
H395Y	-0.333	Neutral			

```
#!/usr/bin/perl
use strict;
use Bio::SeqIO;
my %unique;
my $file   = "both.fasta";
my $seqio  = Bio::SeqIO->new(-file => $file, -format => "fasta");
my $outseq = Bio::SeqIO->new(-file => ">$file.out", -format => "fasta");

while(my $seqs = $seqio->next_seq) {
  my $id  = $seqs->display_id;
  my $seq = $seqs->seq;
  unless(exists($unique{$seq})) {
    $outseq->write_seq($seqs);
    $unique{$seq} +=1;
  }
}
```

Figure S-1 – Perl script, RemoveRep2.pl used to extract unique aaaA sequences.

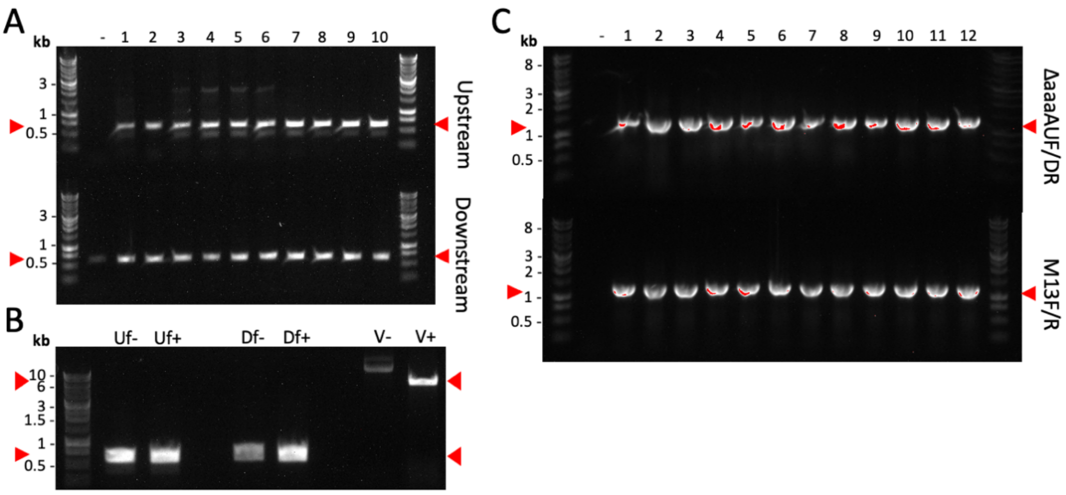

Figure S-2: Cloning for construction of **pEX18Gm::*ΔaaaA suicide vector, using restriction-based cloning. Panel A*** *shows an agarose gel of gradient PCR amplicons of upstream and downstream regions either side of* aaaA. *Lanes are different Tm temperatures in °C: 1. 48.6, 2. 56.3, 3. 52.8, 4. 55.9, 5. 58.9, 6. 62.1, 7. 65.1, 8. 68.1, 9. 70.7, 10. 72.4. Fragment 8 (Tm 68.1) for both were taken forward.* ***Panel B*** *shows an agarose gel of products from restriction digests with XbaI, BamHI and EcoRI.* ***Panel C*** *shows an agarose gel of colony PCR products from 12 transformed colonies (numbered lanes). Expected product: 1341bp with M13F/R and 1272 bp with ΔaaaA-Rx-UpFrag-F/ΔaaaA-Rx-DnFrag-R. Abbreviations: NTC- no template control, Uf – upstream fragment, Df- downstream fragment, V- Vector, +/- cut/uncut. Lane labelled – in panel A and C indicates a no-template PCR control.*

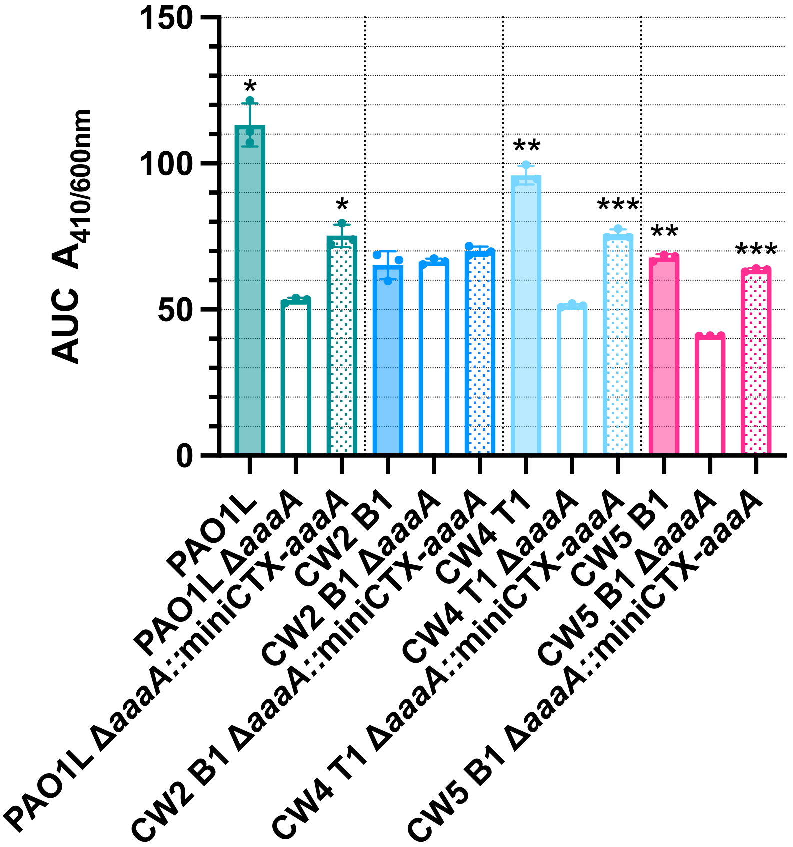

Figure S-3 – AaaA activity decreases in ΔaaaA mutants and is restored in complementation strains, with the exception of CW2 B1, in LB media. *L-arginine-p-nitroanilide cleavage by AaaA in PAO1L, CW2 B1, CW4 T1, and CW5 B1 WT, ΔaaaA and complementation strains measured over 24 hrs following growth in LB. Absorbance was measured at 410 nm. Significant differences were determined by Brown-Forsythe and Welch's ANOVA, with Dunnett's T3 multiple comparisons comparing each group to their respective ΔaaaA mutant. *p<0.05, **p<0.01, ***p<0.001. n=3 repeats, mean ± SD bars.*

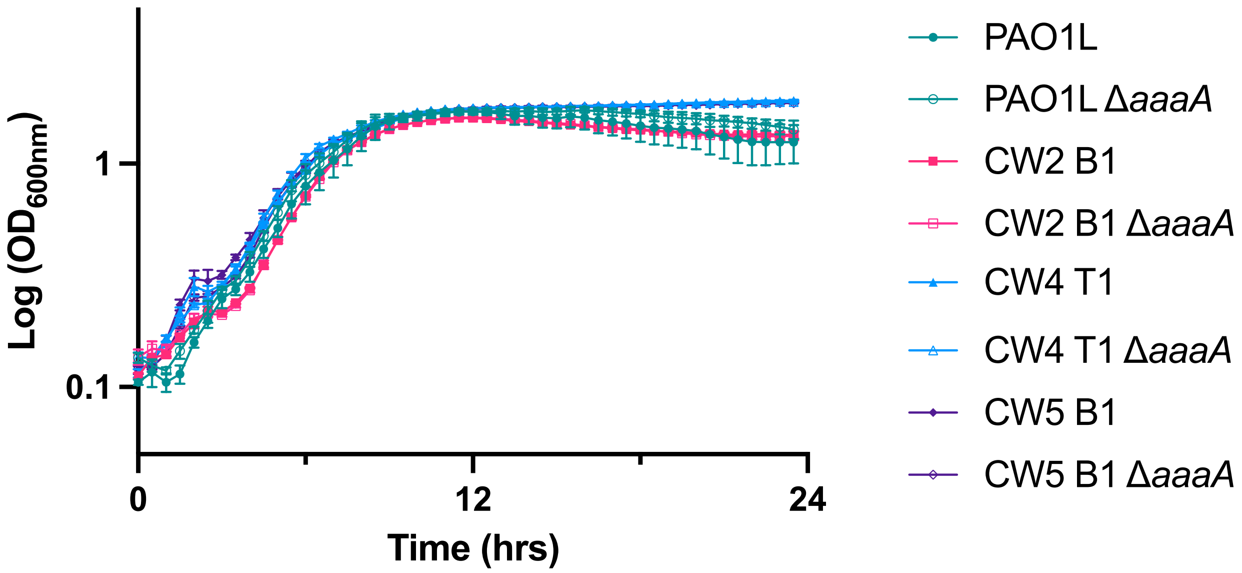

Figure S-4 – Growth kinetics of *P. aeruginosa* WT and Δ*aaaA* strains in LB medium. Growth curves of PAO1L and clinical isolates (CW2 B1, CW4 T1, CW5 B1) and their corresponding Δ*aaaA* mutants were monitored in LB medium using a plate reader. Optical density (OD_{600}) was measured every 30 min for 24 hrs. n=3 repeats, mean ± SD bars.

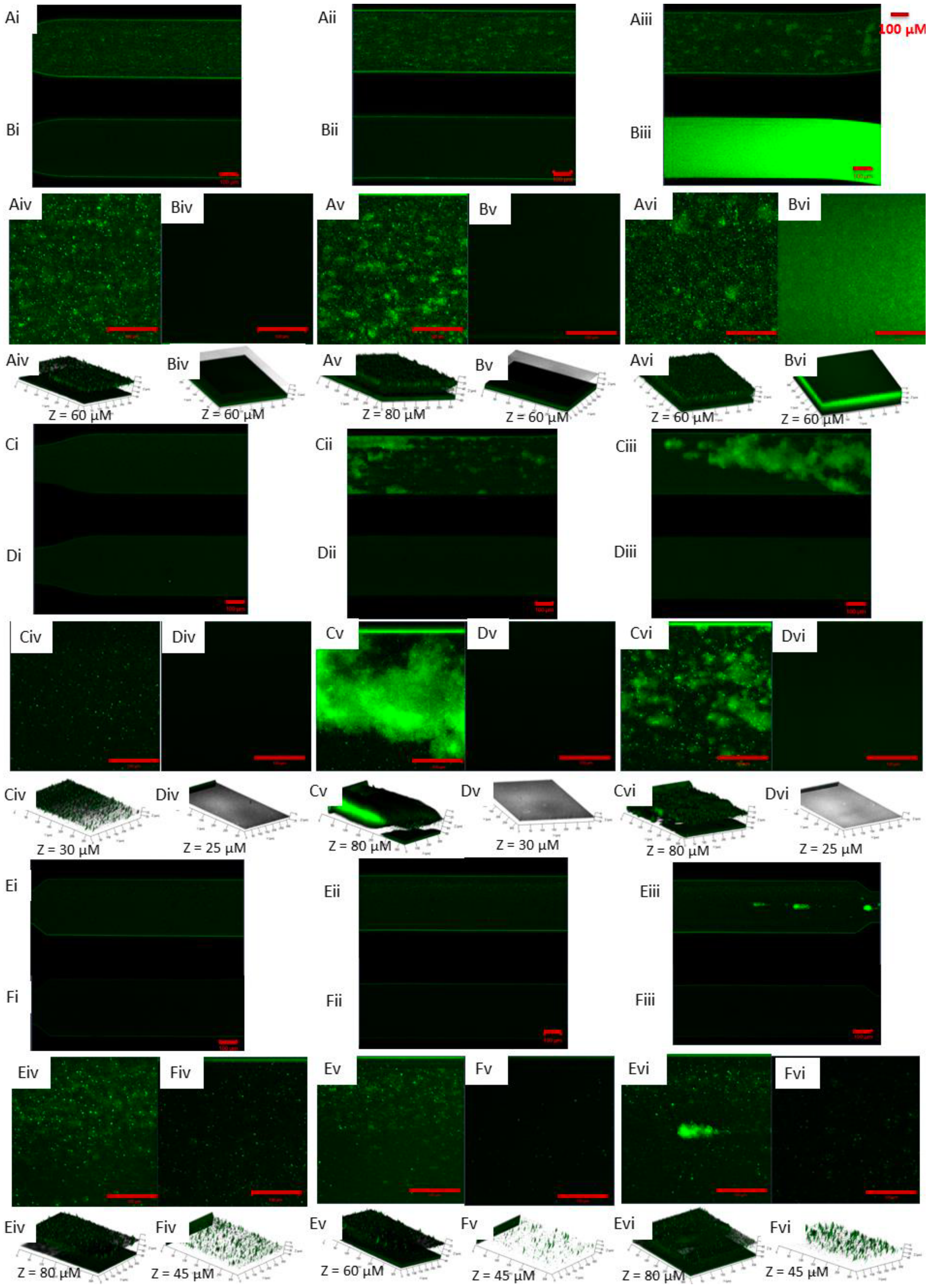

Figure S-5 – LESB65 forms biofilms that are reduced in thickness upon deletion of aaaA in a microfluidic system (Bioflux™). *Biofilms were grown in ASM at 37°C for 15 hrs aerobically. Confocal microscopy was taken at end point. SYTO 9 and PI were added to media in the inlet. Each letter refers to a separate flow cell containing a bacterial strain: A, C, E – LEBS65 WT, B, D, F – LESB65 ΔaaaA. Each pair (e.g., A and B) were tested on the same day. Roman numerals refer to the picture replicate number within the same flow cell. A picture was taken at the left side of the channel at 5x magnification (i), centre 5x (ii), right 5x (iii), a z-stack at 20x left (iv), z-stack at 20x centre (v), z-stack at 20x right (vi).*
